# Components of the *E. coli *envelope are affected by and can react to protein over-production in the cytoplasm

**DOI:** 10.1186/1475-2859-8-32

**Published:** 2009-06-05

**Authors:** Riccardo Villa, Marina Lotti, Pietro Gatti-Lafranconi

**Affiliations:** 1 Dipartimento di Biotecnologie e Bioscienze, Università degli Studi di Milano-Bicocca, Piazza della Scienza 2, Milano, Italy; 2 Department of Biochemistry, University of Cambridge, 80 Tennis Court Road, Cambridge, CB2 1GA, UK

## Abstract

**Background:**

Protein over-expression in bacteria is still the easiest, cheapest and therefore preferred way to obtain large amounts of proteins for industrial and laboratory scale preparations. Several studies emphasized the importance of understanding cellular and molecular mechanisms triggered by protein over-production in order to obtain higher yield and better quality of the recombinant product. Almost every step leading to a fully functional polypeptide has been investigated, from mRNA stability to the role of molecular chaperones, from aggregation to bottlenecks in the secretory pathway. In this context, we focused on the still poorly addressed relationship between protein production in the cytoplasm and the bacterial envelope, an active and reactive cell compartment that controls interactions with the environment and several major cellular processes. Results available to date show that the accumulation of foreign proteins in the cytoplasm induces changes in the membrane lipids and in the levels of mRNAs for some membrane proteins. However, a direct connection between membrane protein expression levels and soluble/aggregated protein accumulation in the cytoplasm has never been reported.

**Results:**

By the use of a combined physiological and proteomic approach, we investigated the effects on the cell membrane of *E. coli *of the overexpression of two recombinant proteins, the *B. cepacia *lipase (BCL) and the green fluorescent protein (GFP). Both polypeptides are expressed in the cytoplasm at similar levels but GFP is fully soluble whereas inactive BCL accumulates in inclusion bodies.

Growth and viability of the transformed cells were tested in the presence of different drugs. We found that chloramphenycol preferentially inhibited the strain over-producing GFP while SDS was more effective when BCL inclusion bodies accumulated in the cytoplasm. In contrast, both proteins induced a similar response in the membrane proteome, i.e. increased levels of LamB, OmpF, OmpA and TolC. Under all tested conditions, the lipopolysaccharide was not affected, suggesting that a specific rather than a generalized rearrangement of the envelope was induced.

**Conclusion:**

Taking together physiological and biochemical evidence, our work indicates that the *E. coli *envelope can sense protein over-expression in the cytoplasm and react by modulating the abundance of some membrane proteins, with possible consequences on the membrane traffic of small solutes, i.e. nutrients, drugs and metabolites. Such a response seems to be independent on the nature of the protein being over-expressed. On the other hand both our data reported herein and previous results indicate that membrane lipids may act as a second stress sensor responsive to the aggregation state of the recombinant protein and further contribute to changes in cellular exchanges with the environment.

## Background

Despite a number of new hosts developed in the latest years, bacterial cells remain the preferred system for the efficient and cheap manufacturing of recombinant proteins for both research and industrial production. For this reason, several recent reports addressed topics of relevance for the successful expression in *Escherichia coli *cells, such as the fine tuning of protein solubility [1-4], the transcriptional response induced by over-production [5], the effect of chaperones and proteases [6-10] and the complexity of *in vivo *aggregation [3,11-13]. Moreover, strategies for the heterologous expression of membrane proteins have been developed [14]. The amount of detailed information available and the continuous demand for new proteins to be efficiently produced are indicating that the time for descriptive papers is (almost) over. There is a pressing need for a better rationalisation of the production process that includes, for any specific protein to be expressed, yields, host physiology and the process of *in vivo *folding and aggregation. In this conceptual frame, the recognition that protein over-production is a cause of stress for cells suggested that an in-depth understanding of such phenomena is necessary to successfully exploit microbial factories [15]. However, although the already cited papers represent excellent investigation of the bases, consequences and causes of protein aggregation in the cytoplasm, a variety of aspects has not been addressed yet.

The cytoplasm is a bacterial compartment actively (and passively) interacting with all others, modifying itself and the whole cell according to internal and external stimuli. We focused on the critical relation between protein production in the cytoplasm and the bacterial envelope, still scarcely investigated also due to technical challenges related to the study of membrane proteins. Membrane proteomics in fact is still a major bottleneck, despite the development of powerful bioinformatics [16] and high-throughput approaches [17,18]. Membranes are the major cell defence against drugs, pathogens or adverse environmental conditions and are widely recognized to be involved in most cellular responses induced by a variety of stress causes [19,20]. Alba and Gross demonstrated that an alternative sigma factor, σ^24^, is responsible for the activation of specific genes involved in membrane biogenesis and lipopolysaccharide (LPS) biosynthesis and that its activity can be triggered by protein misfolding in the periplasm [21]. It has also recently been shown that the periplasm is a reactive cellular compartment where protein aggregation is strongly disfavoured [22,23]. Lipids, regarded in the past as the bare components of membrane bilayers, are now considered as a functional stress sensor able to influence gene expression and stress response in the cytoplasm of both prokaryotic and eukaryotic organisms [24,25]. Moreover, a recent study provided the first experimental evidence that the lipid moiety of the bacterial membrane is affected by the aggregation state of recombinant proteins expressed in the cytoplasm [26]. Evidence suggests that there might be a correlation between cytoplasmic protein accumulation and bacterial envelope rearrangements.

By means of a combined physiological and proteomic approach, we have investigated the effect of soluble and insoluble proteins expressed in the cytoplasm of *E. coli *on some properties of the bacterial membrane. We monitored the growth of strains over-expressing model proteins in the presence of different drugs to evaluate their ability to counteract cytoplasmic or membrane damages. We also addressed alterations in LPS structure or quantity and tried to connect these findings with the membrane proteome. Our results indicate the existence of overlapping rearrangements induced in the cell membrane by protein over-expression, a generic one related to protein overload in the cytoplasm and a second response that appears to be specific for the nature of the protein. Far from being exhaustive, this report is bridging studies on *in vivo *aggregation with bacterial physiology, host strains development and the membrane relevance in cellular regulations and provides a direct description of membrane rearrangements triggered by protein accumulation in the cytoplasm.

## Methods

### DNA manipulation, bacterial strains, growth media and chemicals

*E. coli *strain DH5α was used for standard cloning procedures while expression was carried out in the BL21 (DE3) strain (Invitrogen, US). The synthetic *Burkholderia cepacia *lipase (BCL) gene was designed based on the protein sequence corresponding to the 3LIP crystal structure [27], codon optimized for expression in *E. coli *and synthesized at Genscript (US). Both BCL and the green fluorescent protein (GFP) were expressed from the pET-19b plasmid (Novagen, US). Standard growth medium was 0.5% NaCl Luria broth [LB] supplemented with 100 μg/ml ampicillin [LB-amp] and agar (18 g/l), when requested. Protein production was carried out as follows, if not differently stated: over-night cultures derived from single-colonies were used to inoculate 300 mL of LB-amp, cultures were grown at 37°C until OD_600 _≥ 0.2, then shifted at 30°C for 30 minutes and finally induced with 0.1 mM IPTG (isopropyl-beta-D-thiogalactopyranoside). Growth and production of recombinant proteins were monitored for up to 24 hours. Presence of lipolytic activity was assayed by growing all strains on solid LB medium containing IPTG and 1% (v/v) tributyrin [28,29]. Chemicals of analytical grade were purchased from Sigma-Aldrich (US).

### Extraction of proteins and LPS

Cell lysis was achieved by repeated cycles of sonication keeping the cell suspension in ice through the whole process. 200 μl aliquots of the lysate were used to extract total proteins by trichloroacetic acid at a final concentration of 8%. The solution was briefly mixed, ice-incubated for 10 minutes and then centrifuged at 800 × *g *for 10 minutes. The pellet was re-suspended in loading buffer 1× (25 mM Tris-HCl, 5% glycerol, 1% sodium dodecyl sulfate (SDS), 0.5% β-mercapto-ethanol, 0.02% BBF, pH 6.8) and the pH adjusted with Tris-base 1 M. The leftover after sonication was centrifuged at 4°C for 30 minutes at 16000 × *g*, obtaining a supernatant with the soluble protein fraction and a pellet from which aggregates were extracted as reported [30]. In cells over-expressing BCL, soluble and aggregated protein fractions were tested for lipase activity with p-nitrophenyl-laurate (Sigma) and substrate accumulation measured by 405 nm absorbance [28]. All samples were denatured at 99°C for 4 minutes prior to loading on 12% SDS-polyacrylamide gels. LPS were extracted at different time points along the production process as reported in [31] and silver-stained with the LPS-specific protocol as follows. Gels were fixed over-night with 25% (v/v) 2-propanol, 7% acetic acid solution under shaking. Oxidation was induced with 2.7% 2-propanol plus 0.7% (w/v) periodic acid for 5 min. After washing with _dd_H_2_O four times 30 minutes each, gels were incubated with the silver stain solution (20 mM NaOH, 0.7% (w/v) silver nitrate, and 0.2% ammonium hydroxide) for 10 minutes, then washed with _dd_H_2_O four times 10 minutes each. Gels were kept in the developer solution (0.5% (w/v) citric acid, 0.05% formaldehyde), staining stopped at the appropriate time with 0.35% acetic acid and gels eventually conserved in _dd_H_2_O.

### Resistance to drugs

Cultures were harvested two hours after induction with IPTG, equal concentrations of cells incorporated into soft agar (5 g/l) and poured over standard LB-amp agar plates. Growth inhibition was obtained by positioning on the soft agar layer three blotting paper disks soaked in SDS (2 mg), Chloramphenycol (200 μg) or Rifampicin (200 μg). To measure cell viability on drug-containing plates, volumes equivalent to 1.5 OD_600 nm_/ml were withdrawn from the cultures two hours after IPTG induction (and at the same time point for non induced samples) and spotted on LB-amp agar plates as serial 1:10 dilutions. Experiments were carried out in duplicate on plates +/- IPTG (0.05 mM) and one of the following drugs: SDS (2 mg/ml), Rifampicin (0.1 mg/ml) or Chloramphenycol (0.1 mg/ml). Statistical significance was assessed by running a comparative t-test between each set of measurements and control data.

### Extraction of membrane proteins

Cells were harvested 4 hours after induction and re-suspended in a cold solution of 10 mM Tris-HCl, 0.75 M sucrose (pH 7.8) containing 300 μl of lisozyme (100 μg/ml in 1.5 mM EDTA pH 7.5) to prepare spheroplasts. Two volumes of a cold 1.5 mM EDTA solution (pH 7.5) were slowly added by a peristaltic pump, carefully pouring the buffer just under the liquid surface, and samples kept 10 min in ice with soft stirring. 10 μM PMSF and 0.2 mM DTT were added, samples sonicated and the supernatants collected by centrifugation at 1400 × *g *at 4°C for 20 minutes. Pellets containing whole membranes were recovered after ultracentrifugation of the supernatant at 360000 × *g *for two hours (SORVALL Discovery 90SE, T890 rotor) and re-suspended in 350 μl of 3.3 mM Tris-HCl, 0.3 mM EDTA, 0.25 mM sucrose (pH 7.8). Buffer was exchanged overnight against 2 L of 33 μM Tris-HCl, 3 μM EDTA, 0.25 mM sucrose solution (pH 7.8). A second ultracentrifugation step in presence of solubilization buffer SB (7 M Urea, 2 M Thiourea, 4% CHAPS) was finally performed. To solubilize extracts, pellets were re-suspended in 150 μl of SB buffer, kept at RT for two hours under shaking and then centrifuged at maximum speed for 20 minutes at 4°C. Supernatants were harvested, added of 4 volumes of cold acetone and inverted repeatedly. Samples were kept at -20°C for 1.5 hours to increase protein precipitation yield, centrifuged at maximum speed at 4°C for 20 minutes and air dried over-night.

### Two Dimension Electrophoresis

Extracts of membrane proteins were re-suspended in 150 μl of SB buffer, quantified by the Bradford assay method and the volume containing 200 μg protein diluted in 300 μl of SB with 0.33 mM EDTA, 60 mM DTE, 60 mM Iodoacetamide and 1% carrier ampholytes. After 30 minutes at room temperature samples were loaded on the IEF apparatus (BIO-RAD PROTEAN IEF Cell) and hydrated over-night. Focusing was carried out using intensities in the 200–8000 V range for appropriate time periods. IEF strips were re-equilibrated in 7 ml buffer RB (6 M Urea, 2% SDS, 30% glycerol, 50 mM Tris-HCl, pH 6.8) in presence of 2.5% DTE or DTT for 20 min under shaking, then in RB with 2.5% IAA. Gel strips were adjusted on the top of an 11% SDS-polyacrylamide gel and incorporated with 0.5% agarose. PAGE was carried in a refrigerated system (Ettan DALTsix electrophoresys unit, Amersham Biosciences) at 15 to 30 mA. Gels were finally stained with GelCode Blue Stain Reagent (Pierce).

### Spot matching and identification

The Progenesis SameSpot software (Nonlinear Dynamics, US) was used for gel alignments and spot matching. Spots corresponding to proteins identified by mass spectrometry (see below) were selected and the intensity of manually refined spot areas automatically measured. Values were normalized for each gel staining efficiency, averaged for replicates and finally referred to the control strain. Expression changes were considered significant if the p-value of the corresponding spot provided by the program (running an Anova test) was lower or close to 0.05. Several spots were cut, trypsin digested and proteins identified by nano-ESI-MS on a hybrid Quadrupole-Time-of-Flight mass spectrometer (QSTAR ELITE, Applied Biosystems, Foster City, CA, USA). All proteins listed were identified using the MASCOT software with probability scores above threshold. Only expression changes fulfilling statistical significance requirements were considered in results and discussion.

## Results

### Expression of the test recombinant proteins

**Figure 1:**
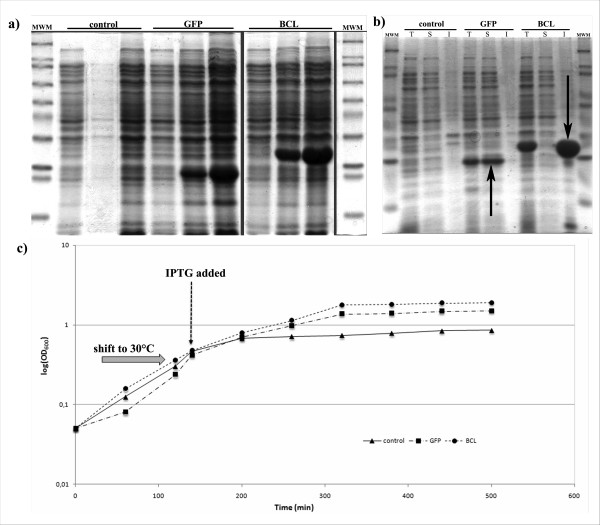
**a) Total proteins of strains grown at 30°C extracted at 0, 2 h and 6 h after induction**. b) Total [T], soluble [S] and aggregated [I] protein fractions extracted from recombinant strains 4 hours after IPTG addition. Arrows indicate the position of the recombinant protein. c) Growth profile of the three strains used in this study. After growth at 37°C, cultures were shifted to 30°C for 30 minutes prior to 0.1 mM IPTG addition.

The well-characterized *E. coli *strain BL21 (DE3) was transformed with two plasmids bearing the GFP and BCL coding sequences. Both proteins are produced without any extra tag. A third strain carried the empty pET19b vector as a control. The system was designed to minimize additional effects due to the over-expressed proteins: GFP and BCL have similar sizes, no obvious biological activity in the host cell and are produced at comparable level (Figure 1a).

Expression at 30°C resulted in GFP and BCL completely partitioned in the soluble and in the insoluble protein fraction, respectively (Figure 1b). It is known that aggregated proteins may retain residual catalytic activity [12]. Therefore we checked for activity of aggregated BCL that might interfere with the analysis of results by raising additional cell responses. We did not evidence any lipase activity neither in the transformed strains [see additional file 1] nor in preparations of purified inclusion bodies (not shown) in agreement with the strict requirement of BCL for a specific folding chaperone lacking in *E. coli *cells [32,33]. Therefore, in the following BCL was used as a reporter for the effects of the accumulation of inclusion bodies on the cell envelope while GFP was expected to elicit the response related to the over-production of soluble proteins.

Growth and expression at 30°C provided the optimal condition for our experimental setup, in terms of protein production, aggregation and growth rate. However, since changes in temperature might induce transient physiological rearrangements, we delayed IPTG addition to uncouple protein synthesis to the temperature shift. By applying the same procedure to all strains, all possible effects due to temperature changes have been normalized and thus excluded from the analysis. Growth kinetics revealed no major differences among strains during the first two hours after induction (Figure 1c), this time point being therefore selected as the default condition for all subsequent physiological studies.

### Effects of the expression of recombinant proteins on cell sensitivity to drugs

To highlight possible effects of protein accumulation on bacterial membranes, initially we monitored cell resistance to rifampicin, chloramphenicol and SDS in a growth inhibition experiment. Drugs and drugs concentrations were selected on the basis of information reported in the literature [34] and further optimized for the specific strains under study to obtain clear and measurable halos (not shown). The first two drugs are antibiotics whose activity is expressed at the cytoplasmic level (on RNA polymerase and the 50S ribosomal subunit, respectively) and must therefore be transported or diffuse across membranes [35], while SDS damages membranes by direct interaction, affecting cell growth and viability [36]. Induced bacterial cultures were plated by the soft-agar technique on Petri dishes containing the three drugs, plates were incubated at 30°C over night and inhibition halos measured (Table 1). Rifampicin is believed to diffuse across the outer membrane *per se*, without being significantly affected by the nature and composition thereof [35]. Accordingly, we did not observe important differences among strains (Table 1). The major increase in CAF sensitivity of the cells expressing GFP could reflect an easier diffusion of the drug inside the cell or the impairment of the translational machinery in these transformed cells. On the other hand, the reverse behaviour of the two recombinant strains upon SDS treatment indicates that damages at the membrane have more severe effects or are more slowly repaired in the BCL-expressing strain. Interestingly, the observation that BCL and GFP elicit opposite effects suggests that the solubility state of the over-expressed protein and not its presence affects the ability to counteract drugs. In this view, the higher resistance of GFP-expressing cells to SDS treatment suggest a protective role conferred by the specific stress response elicited by the over-expression of this particular protein.

**Table 1:** Effects on drug resistance.

	*Rifampicin*		*Chloramphenicol*		*SDS*	
	*mm*	*%*	*mm*	*%*	*mm*	*%*

**Control**	26.3 ± 0.6		36.0 ± 2.6		12.3 ± 1	
**GFP**	28.3 ± 1.5	+7.6 ± 0.4	^#^47.0 ± 1.0	+30.6 ± 2.3	^#^10.0 ± 0	-18.9 ± 2
**BCL**	27.3 ± 0.6	+3.8 ± 0.1	37.3 ± 0.6	+3.7 ± 0.2	^#^15.7 ± 1	+27.0 ± 4

Raw halo measurements (mm) and changes relative to control (%) are reported for experiments run in triplicate. Independent experiments were run and, although different batch of plates gave different absolute values (mm), relative changes were highly reproducible (not shown). Negative numbers indicate higher resistance, positive increased sensitivity. Data marked by ^# ^indicates differences with control values showed statistical significance (t-test p ≤ 0.05). The following amounts of drugs were used: RIF 200 μg, CAF 200 μg, SDS 2 mg.

**Figure 2:**
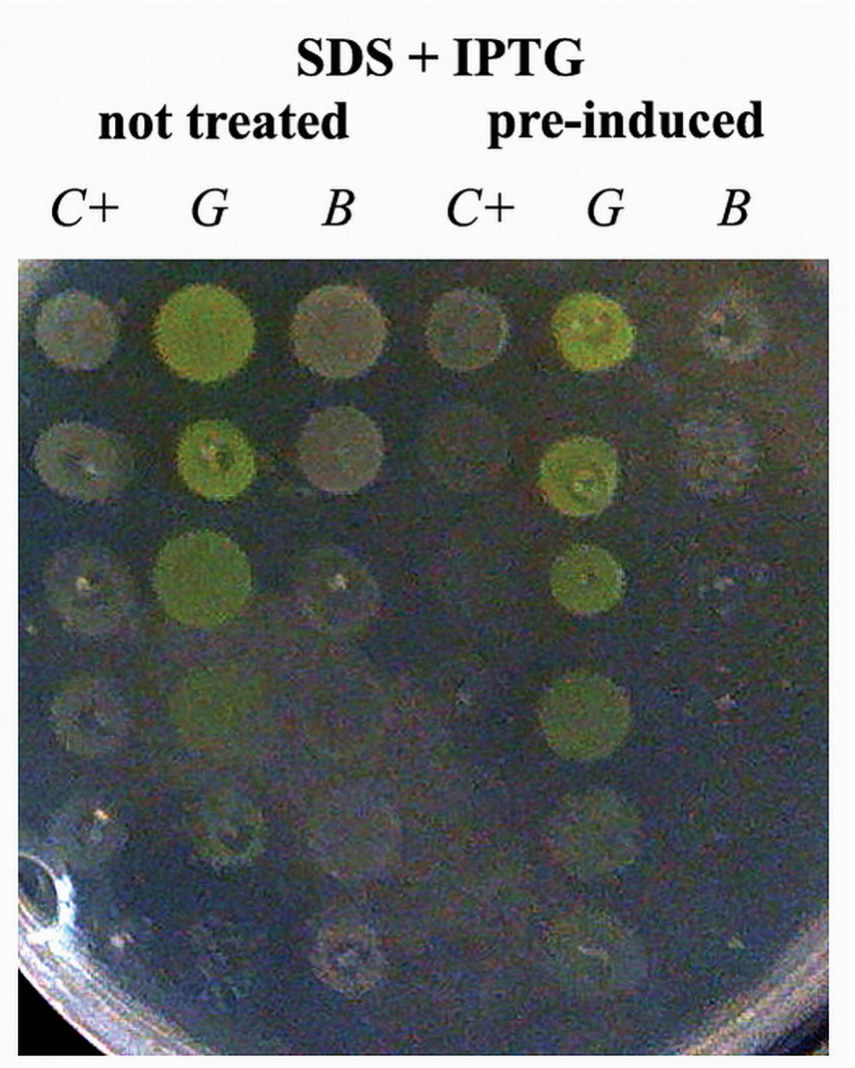
**Recombinant cells (C+: control; G: GFP; B: BCL) plated on an IPTG- and SDS-containing Petri dish**. The first three columns correspond to non-pre-induced cultures; the last three to cultures induced with 0.1 mM IPTG. Spots range between pure cultures to 5-fold dilutions thereof by means of 1:10 steps (top to bottom). The accumulation of active GFP causes cells to assume a greenish yellow colour.

Cell viability was further assessed with a different experimental procedure [34,37] with the aim of checking the direct involvement of protein over-expression on growth inhibition. In this experiment, cultures were spotted on solid medium containing IPTG after a 2-hour pre-induction in liquid medium or, as controls, after 2 hours in LB without IPTG (differences in growth are not evident at this stage, see Figure 1c). All strains are thus plated in the same condition (presence of the inducer) while differing in background (presence/absence of the over-expressed protein in the cytoplasm). Results indicated that, while antibiotics did not produce significantly different effects (not shown), in the presence of SDS the growth of control cells and of those expressing BCL – but not that of GFP producers – was severely impaired only in cultures exposed to IPTG before spotting (Figure 2). The toxicity of empty vectors in the specific case of BL21 cells has already been reported [38] and we also verified, in control experiments, that the lone protein production due to the presence of the inducer in plates was not the cause of toxicity and that the pre-induction step *per se *had no effect on the SDS-sensitivity [see additional file 2]. Thus, the pre-incubation step is the critical factor generating in the control and BCL-producing strains a different physiological background than in GFP-cells. This difference leads to opposite behaviours during the expression phase when cells are grown on SDS (but not in control plates or in the presence of antibiotics). Considering the nature of the drug, membrane must be directly or indirectly affected by the solubility state of the proteins accumulating in the cytoplasm.

### Lipopolysaccharide isolation and analysis

The lipopolysaccharide (LPS) is an essential component of the *E. coli *outer membrane greatly contributing to the structural integrity of the bacteria. In fact, it provides defence against many toxic compounds and its lack causes cell death [39]. Modifications in the structure and expression of LPS have already been reported to be consequences of adaptation processes or to happen as a result of mutagenesis [40]. We therefore extracted LPS from all strains prior and after induction. The nature of the core lipopolysaccharide did not change during recombinant protein expression although its concentration did (data not shown). This behaviour, however, occurred in all strains enclosed the control so LPS is not likely to be affected by protein over-expression in the cytoplasm.

### Membrane proteins

**Figure 3:**
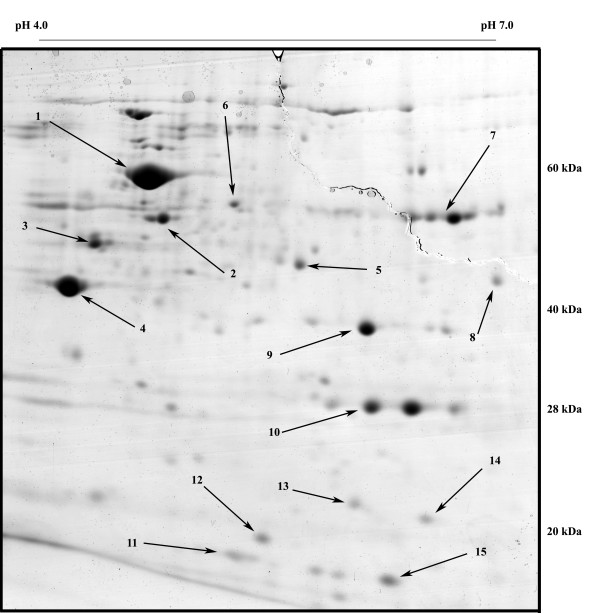
**2-DE gel of membrane proteins extracted from the GFP over-producing strain with relevant spots indicated**. Numbers refer to TABLE 2.

The current view of the dynamics of bacterial membranes pictures complex interactions between lipids and proteins, such that changes in one should cause rearrangements in the other component as well. Since rearrangements in the lipid moiety were reported to be a consequence of protein over-expression [26], we investigated possible changes in composition or levels of expression of membranes protein components. Membrane extracts were loaded on IEF strips (pH range 4–7) and then separated on 11% SDS-polyacrylamide gels. To minimize variability, gels were run in duplicate using extracts from independent fermentations. Separation was tuned to enhance spots in the pI range 4.5–6.0 and mass range 60 to 25 kDa [see additional file 3]. After separation it was possible to identify several membrane and periplasmatic proteins (OmpA, OmpF, OmpX, LamB, TolC, TolB), inner membrane-bound proteins (ATPB, DLDH, PTNAB, EF-Tu) and others polypeptides often found in such extracts due to their high concentration or propensity to interact with other proteins or membranes (GroEL, FTNA, IbpA, RS6, DPS, SSB). An example of a typical gel is shown in Figure 3, where arrows indicate the most relevant spots then selected for protein identification. It is noteworthy that most identified proteins are related to stress responses. These experiments revealed an interesting pattern: GFP- and BCL-over-producing strains showed 2 to 5 time higher expression of several major membrane proteins with respect to the control (Table 2). There are, however, no strong indications for a differential regulation of membrane proteins as a consequence of the aggregation state of the heterologous protein, apart from small but indicative changes in specific membrane proteins (cfr. OmpA and OmpF).

**Table 2:** Intensity changes of selected spots identified from 2-DE gels. Numbers refer to Figure 3.

***n°***	***name***	***Anova (p)^a^***	***function***	***ratio BCL***	***ratio GFP***	***references***
3	LamB	0.00017	maltoporin	3.8	4.7	[35,50,51]
4	OmpF	0.014	outer membrane protein F	3.0	2.0	[35,36,53]
9	OmpA	0.008	outer membrane protein A	1.7	2.4	[26,35,49]
6	TolC	0.076	outer membrane TolC	2.0	2.0	[46,52]

5	EF-Tu	0.015	Elongation factor Tu	1.3	0.9	
1	GroEL	n.a.	HSP 60	1.1	0.9	
7	DLDH	n.a.	Dihydrolipoyl dehydrogenase	1.0	0.9	
2	ATPB	>>	ATP synthase subunit beta			
8	TolB	>>	Protein tolB			
10	GFP	>>	over-expressed GFP			
11	RS6	>>	30S ribosomal protein S6			
12	OmpX	>>	outer membrane protein X			
13	SSB	>>	single-stranded DNA binding protein			
14	DPS	>>	DNA protection during starvation protein			
15	IbpA	>>	small heat shock protein IbpA			

Ratios were calculated from the normalized spot intensities averaged for gel replicates and finally expressed as BCL- or GFP-over-Control ratios. The first block contains membrane proteins affected by over-expression with statistical significance.

^a ^Probability score calculated by the program taking in consideration all previous parameters. "n.a." means volume was calculated from the sum of all spot intensities belonging to the same protein (as determined by ESI-MS spot identification for multiplets), due to excessive protein amount in gels or poor focusing. ">>" indicate value was much above threshold, no ratio is reported although protein presence was determined by mass analysis.

## Discussion

In 2003 Michael Edidin reviewed the history of studies on lipid bilayers [41]. Although the main focus of the paper was the eukaryotic plasma membrane, some considerations apply to our system as well: the dynamic interplay between membrane components can hardly be captured, information is lacking about traffic to and from membranes as well as about association with other elements (cytoskeleton in the review, periplasmic space, peptidoglycan, LPS in our case). Nowadays it is generally accepted that membranes are a complex, heterogeneous cell compartment that can be described as a network of small dynamic domains where specific proteins cluster together rather than being homogeneously dispersed. More recently, our understanding of membrane complexity was further increased by the identification of lipids as cellular stress sensors [25,42], the definition of a revised protein diffusion/mobility model [43], the identification of genetic modulators of membrane stress responses [21] and by considering membrane-associated peripheral proteins as a functional component of the membrane interaction network [44]. Taken together these studies strongly suggest that the cell membrane fulfil all requirements to sense the stress induced by protein over-expression and can act as a cellular defence by modulating traffic with the environment (by changes in fluidity or porin expression), altering surface properties (biofilm composition, virulence) or controlling cell metabolism (proton pumps, ATP synthesis, glycolysis).

In a previous paper [26] we pointed to membranes as a key factor in sensing misfolding and aggregation of recombinant proteins and we showed that lipids undergo rearrangements depending on the aggregation state of the accumulating protein. We report here that over-expression in the cytoplasm elicits changes in the membrane proteins. Such response however seems not to depend on the specific protein produced. As a major difference among the proteins used in this study is *in vivo *solubility, it would be tempting to speculate that the membrane proteome is not responsive to the aggregation state of the protein. In support to our hypothesis, the extensive investigation reported in [5] excluded the transcription factor σ^32 ^as the genetic controller of membrane rearrangements and did not identify any gene regulated by the alternative transcription factor σ^24 ^(sensitive to membrane protein expression levels and misfolding [19,21]) either.

Results from the proteomic analysis (increased expression of LamB, OmpF, OmpA and TolC in GFP and BCL over-producing strains) are consistent with a number of studies in which such proteins are reported to be involved in natural and induced membrane rearrangements [36,45-47]. OmpA folding and stability are clearly connected with membrane stabilization [26,48] and sub-optimal growth conditions are known to trigger a structural rearrangement driving changes in pore size and metabolites traffic [49]. The maltoporin LamB is induced by maltose but also over-expressed in glucose-limited cultures [50,51] while certain antibiotics induce changes in the expression levels of OmpF, TolC, LamB, DPS and other membrane proteins [52,53]. Moreover, DLDH and OmpA have been shown to accumulate after heterologous protein over-expression [26]. This information suggests that the cell reacts to protein over-expression in the cytoplasm in a generalized and non-specific way by improving the traffic of small solutes, i.e. nutrient uptake.

If the reason why membrane proteins are involved in this specific stress response might be explained by the requirement for an increase in nutrient uptake, a satisfactory explanation of how this happens on the basis of available literature is far from straightforward. The on-plate experiments reported here indicate that, when the SDS-induced stress is applied to over-producing cells, the nature of the heterologous protein affects the ability of cells to counteract the toxic effect. Lipids, the direct target of SDS damage, have already been reported to be involved in the stress response related to the aggregation state of the recombinant proteins [26] and IBs are known to specifically trigger lipid rearrangements [54]. We therefore propose that a second stress response might be induced at lipid level, this one responsive to the aggregation state of the recombinant protein, similar to the varying cytoplasmic stress responses induced by different protein aggregates reported by others [55]. Interestingly, 2-DE analysis revealed that BCL and GFP producing cells (but not BCL and control) share a similar protein expression pattern despite showing opposite SDS sensitivity. This suggests that the two responses, triggered by the same event (protein over-production), are not coordinated by the cell. Finally, the absence of changes at LPS biosynthesis and expression levels indicates a specific rather than a generic rearrangement is induced.

## Conclusion

Our results indicate that the *E. coli *envelope is able to sense and actively react to protein over-expression in the cytoplasm and is thus entitled to be part of the complex network of cellular responses this event triggers. Our results indicate that the aggregation state of the accumulating protein affects the lipid moiety, while membrane proteins undergo a rather non specific reaction pattern. The combination of these events may increase uptake and availability of substrates and metabolites. As the cell envelope modulates trafficking with the environment, it can be easily hypothesized that artificially induced events (heterologous protein accumulation and the presence of antibiotics in the medium) mimic naturally occurring physiological (mutations or metabolic changes) and nutritional (environmental) alterations. Although these rearrangements could be part of a still uncharacterised regulatory pathway, lipids stand out as a critical component as it appears that they are not only involved in envelope rearrangements but might also act as a second, independent sensor for the solubility state of the accumulating protein.

## Abbreviations

LPS: lipopolysaccharide; BCL: *Burkholderia cepacia *lipase; GFP: green fluorescent protein; CAF: chloramphenycol; RIF: rifampicin; SDS: sodium dodecyl sulfate; IEF: isoelectric focusing; PAGE: polyacrylamide gel electrophoresis; 2-DE: two dimension electrophoresis; IBs: inclusion bodies; pI: isoelectric point; MW: molecular weight.

## Competing interests

The authors declare that they have no competing interests.

## Authors' contributions

RV performed *in vivo *assays, LPS and membrane proteins extractions and the proteomic analysis. PG-L designed the project, provided constructs, performed statistical analyses, supervised experimental work and wrote the paper. ML extensively contributed to the writing and supervised the whole project. All authors read and approved the final manuscript.

## Supplementary Material

Additional file 1 **On-plate detection of lipase activity**. LB-agar plate containing 0.1 mM IPTG and 1% (v/v) tributyrin. Strains were grown overnight at 37°C then incubated at 30°C for 24 h. The presence of lipase activity is indicated by a clear hydrolysis halos around cells. A BL21 strain bearing an active lipase (PFL, [30]) was also plated as a positive control. Click here for file

Additional file 2 **Pre-induction is required for SDS-dependent growth impairment**. Spots plated on IPTG-deprived (left) and IPTG-containing (middle) control Petri dishes or in the presence of SDS but without IPTG (right). First three columns of each plate are derived from un-induced fermentations; the last 3 ones from 0.1 mM IPTG induced cultures. Spot range between pure cultures to 5-fold dilutions thereof by means of 1:10 steps. The accumulation of active GFP causes cells to assume a greenish yellow colour. Used abbreviations: C+, control; G, GFP; B, BCL. Click here for file

Additional file 3 **Set of gels used for proteomic analysis**. 2DE-gel pairs in the pI and MW range of interest. Arrows indicate over-expressed proteins. Click here for file
