# Supplementary figures and images for: Components of the E. coli envelope are affected by and can react to protein over-production in the cytoplasm
Source: Microb Cell Fact. 2009 Jun 5;8:32. doi: 10.1186/1475-2859-8-32 (PMC2701923; doi:10.1186/1475-2859-8-32)

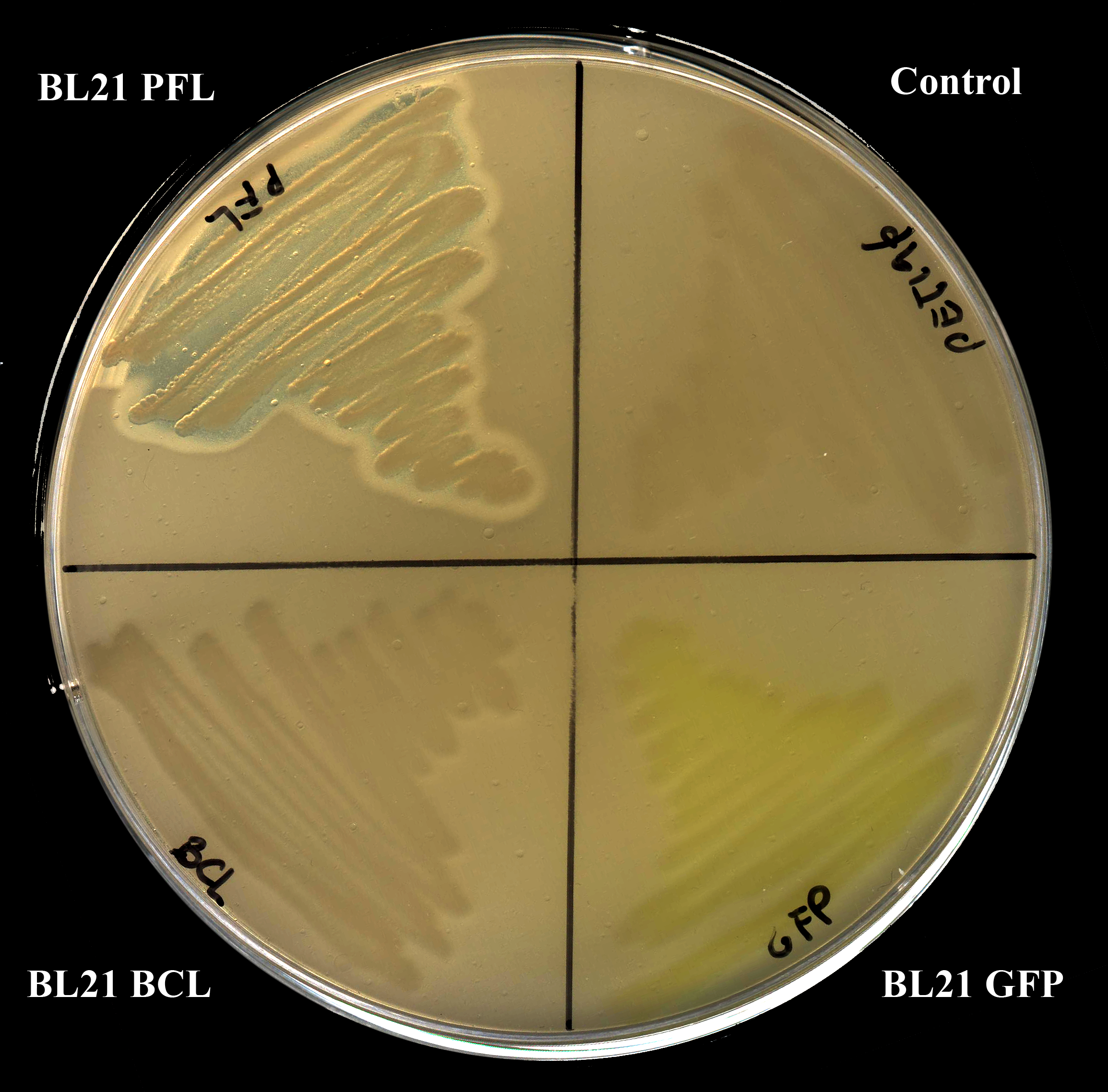

Supplement: Additional file 1 — On-plate detection of lipase activity. LB-agar plate containing 0.1 mM IPTG and 1% (v/v) tributyrin. Strains were grown overnight at 37°C then incubated at 30°C for 24 h. The presence of lipase activity is indicated by a clear hydrolysis halos around cells. A BL21 strain bearing an active lipase (PFL, [30]) was also plated as a positive control. [file 1475-2859-8-32-S1.png]

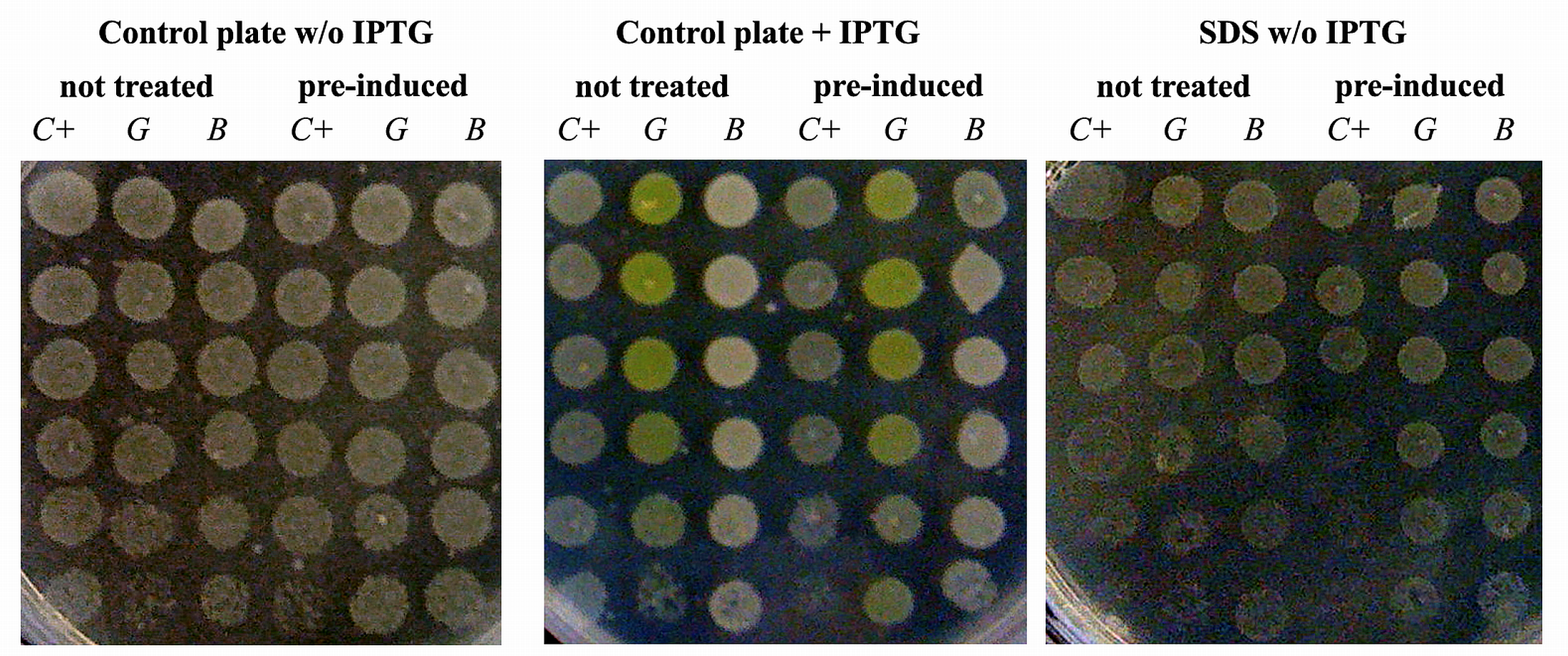

Supplement: Additional file 2 — Pre-induction is required for SDS-dependent growth impairment. Spots plated on IPTG-deprived (left) and IPTG-containing (middle) control Petri dishes or in the presence of SDS but without IPTG (right). First three columns of each plate are derived from un-induced fermentations; the last 3 ones from 0.1 mM IPTG induced cultures. Spot range between pure cultures to 5-fold dilutions thereof by means of 1:10 steps. The accumulation of active GFP causes cells to assume a greenish yellow colour. Used abbreviations: C+, control; G, GFP; B, BCL. [file 1475-2859-8-32-S2.png]

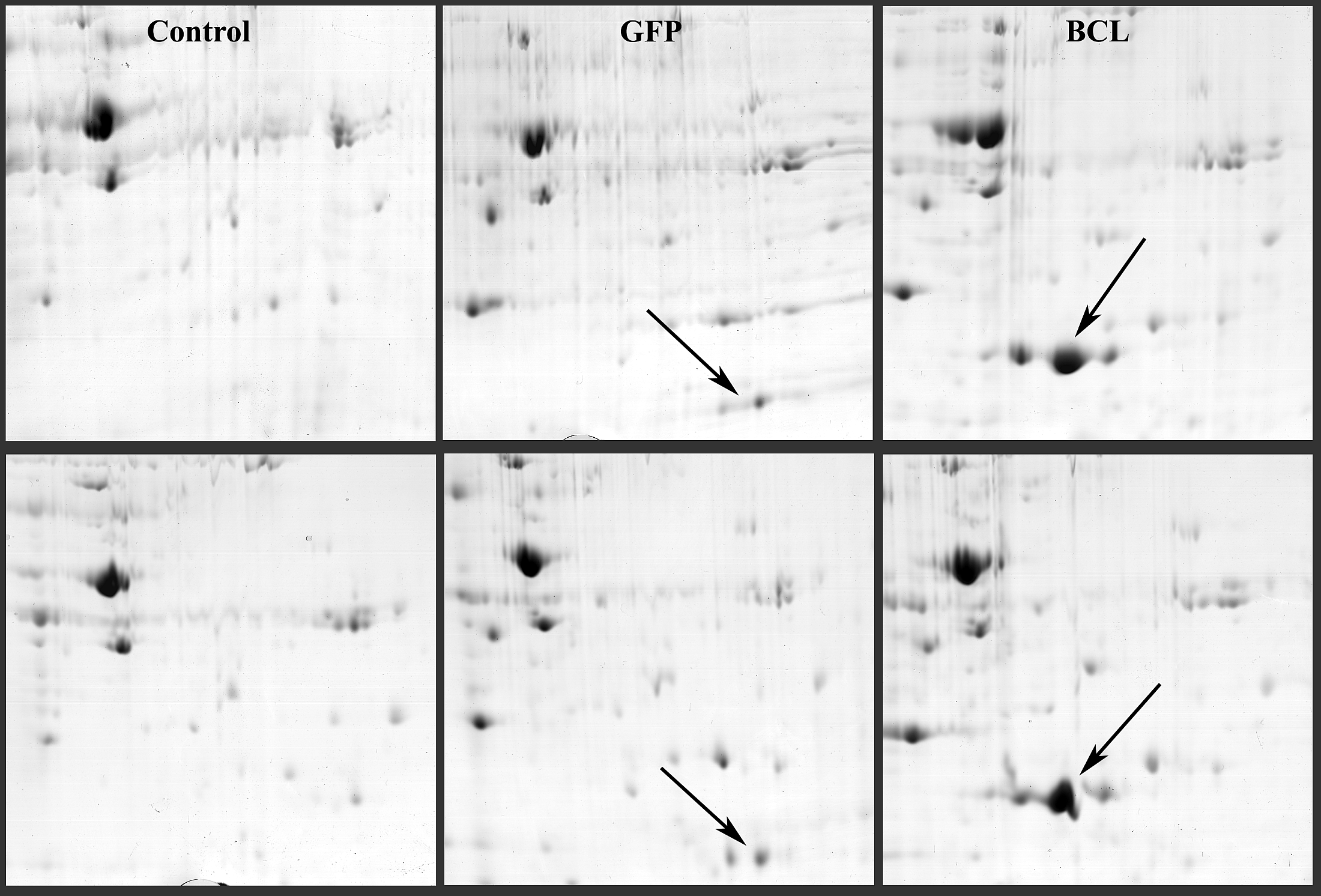

Supplement: Additional file 3 — Set of gels used for proteomic analysis. 2DE-gel pairs in the pI and MW range of interest. Arrows indicate over-expressed proteins. [file 1475-2859-8-32-S3.png]
